# Forecasting of Milk Production in Northern Thailand Using Seasonal Autoregressive Integrated Moving Average, Error Trend Seasonality, and Hybrid Models

**DOI:** 10.3389/fvets.2021.775114

**Published:** 2021-11-30

**Authors:** Veerasak Punyapornwithaya, Katechan Jampachaisri, Kunnanut Klaharn, Chalutwan Sansamur

**Affiliations:** ^1^Veterinary Public Health and Food Safety Centre for Asia Pacific (VPHCAP), Faculty of Veterinary Medicine, Chiang Mai University, Chiang Mai, Thailand; ^2^Research Group for Veterinary Public Health, Faculty of Veterinary Medicine Chiang Mai University, Chiang Mai, Thailand; ^3^Department of Mathematics, Faculty of Science, Naresuan University, Phitsanulok, Thailand; ^4^Bureau of Livestock Standards and Certification, Department of Livestock Development, Bangkok, Thailand; ^5^Akkhraratchakumari Veterinary College, Walailak University, Nakhon Si Thammarat, Thailand

**Keywords:** milk production, forecast, time-series model, hybrid model, decision, Thailand

## Abstract

Milk production in Thailand has increased rapidly, though excess milk supply is one of the major concerns. Forecasting can reveal the important information that can support authorities and stakeholders to establish a plan to compromise the oversupply of milk. The aim of this study was to forecast milk production in the northern region of Thailand using time-series forecast methods. A single-technique model, including seasonal autoregressive integrated moving average (SARIMA) and error trend seasonality (ETS), and a hybrid model of SARIMA-ETS were applied to milk production data to develop forecast models. The performance of the models developed was compared using several error matrices. Results showed that milk production was forecasted to raise by 3.2 to 3.6% annually. The SARIMA-ETS hybrid model had the highest forecast performances compared with other models, and the ETS outperformed the SARIMA in predictive ability. Furthermore, the forecast models highlighted a continuously increasing trend with evidence of a seasonal fluctuation for future milk production. The results from this study emphasizes the need for an effective plan and strategy to manage milk production to alleviate a possible oversupply. Policymakers and stakeholders can use our forecasts to develop short- and long-term strategies for managing milk production.

## Introduction

Thailand's dairy industry is one of the economically important agricultural sectors. In 2018, 1.29 million tons of raw milk were produced in the country (1). According to data from May 2020, there were 24,229 dairy farmers operating dairy farms and the number of dairy cattle in Thailand was 8,11,756 head. The majority of dairy farmers were smallholders who utilized the tie-stall system for farming. The primary breed of dairy cows was Holstein with a small fraction of other breeds. The three most extensive dairy farming regions are central, northeast, and north (2). In the northern region, dairy farms are located in six provinces and the majority of them are in Chiang Mai (3). The average milk production in the north is 437 tons per day (4). Dairy farmers in the north, similar to those in the rest of Thailand, are members of dairy cooperatives that manage milk collection centers (5, 6). Bulk tank milk from dairy farms is collected, cooled, stored at milk collection centers, and then transported to milk processing plants (7).

In recent years, an oversupply of milk has been a matter of concern among policymakers and stakeholders in Thailand (8, 9). Even though the milk supply has risen, the dairy sector struggles to increase milk consumption in the country owing to cultural hurdles (9). In 2020, Thailand's per capita for dairy products was ~17 L per year, which is quite low, compared with other Asian countries such as Japan (31 L per year) and India (49 L per year) (10). The oversupply of milk may pose challenges to several sectors in the supply chain. To control situations of oversupply, authorities and stakeholders need to formulate effective short- and long-term plans to manage milk supply based on accurate estimations of milk production derived from well-accepted prediction methods.

Forecasting is a vital element in economic decision-making. Of the several time-series forecast methods demonstrated in the literature (11–15), the autoregressive integrated moving average (ARIMA) is one of the most commonly used. This method has been extensively used in various domains such as economic (16, 17), human medicine (18–20), veterinary science (21, 22), and agriculture science (23–25). ARIMA is extended by the seasonal autoregressive integrated moving average (SARIMA) method which is suitable for modeling time-series data with a seasonal trend (26–28). Moreover, another commonly used method for analyzing seasonality time-series data is the exponential smoothing (ES) (15, 27). Within a state-space framework, the ES method can combine error (E), trend (T), and seasonal (S) components in a smoothing calculation which is referred to as an error trend seasonality (ETS) model (29). A total of 30 possible ETS combinations within the state-space framework render the ability to analyze any time-series data, even with both heterogeneity and non-linearity (30). Furthermore, in the last two decades, a hybrid modeling which combines forecasting methods has been used in many disciplines to enhance the accuracy of forecast models (11, 15, 31). For example, a study of healthcare data demonstrates that combining SARIMA and ETS (SARIMA-ETS hybrid) methods offers better performance and forecast accuracy than models developed from a single method (32). At present, numerous hybrid models have been proposed; however, selecting methods to be combined as a hybrid model necessitates an understanding of the nature of each time-series data (27). Interestingly, although hybrid models are widely used with a variety of datasets such as medical (33, 34) and environmental data (15, 35), their application to milk production data is very limited.

Several researchers have applied time-series methods to forecast milk production in Bangladesh (36), China (37), Ethiopia (38), and India (39), but the methods used in those studies were limited to ARIMA (11). Although several forecast methods such as artificial neural network (ANN) and support vector machines (SVM) are widely used in time-series models (40, 41), this study focused primarily on the methods that are capable of dealing with seasonality time-series data since milk production is likely to follow a seasonal pattern (39, 42–45). As a result, the SARIMA, ETS, and SARIMA-ETS hybrid models were selected to analyze the milk production data in this study.

Milk production forecasting based on well-accepted statistical methods is essential for government authorities, decision-makers and stakeholders in Thailand to establish short- and long-term plans to deal with future milk production, particularly when an oversupply is anticipated. Hence, the objective of this study was to develop forecast models to predict milk production in northern Thailand using SARIMA, ETS, and SARIMA-ETS hybrid models. Meanwhile, the predictive performances of forecast models were evaluated in order to identify the most accurate model, which could subsequently be used to estimate future milk production.

## Materials and Methods

### Study Area and Milk Production Data

**Figure 1:**
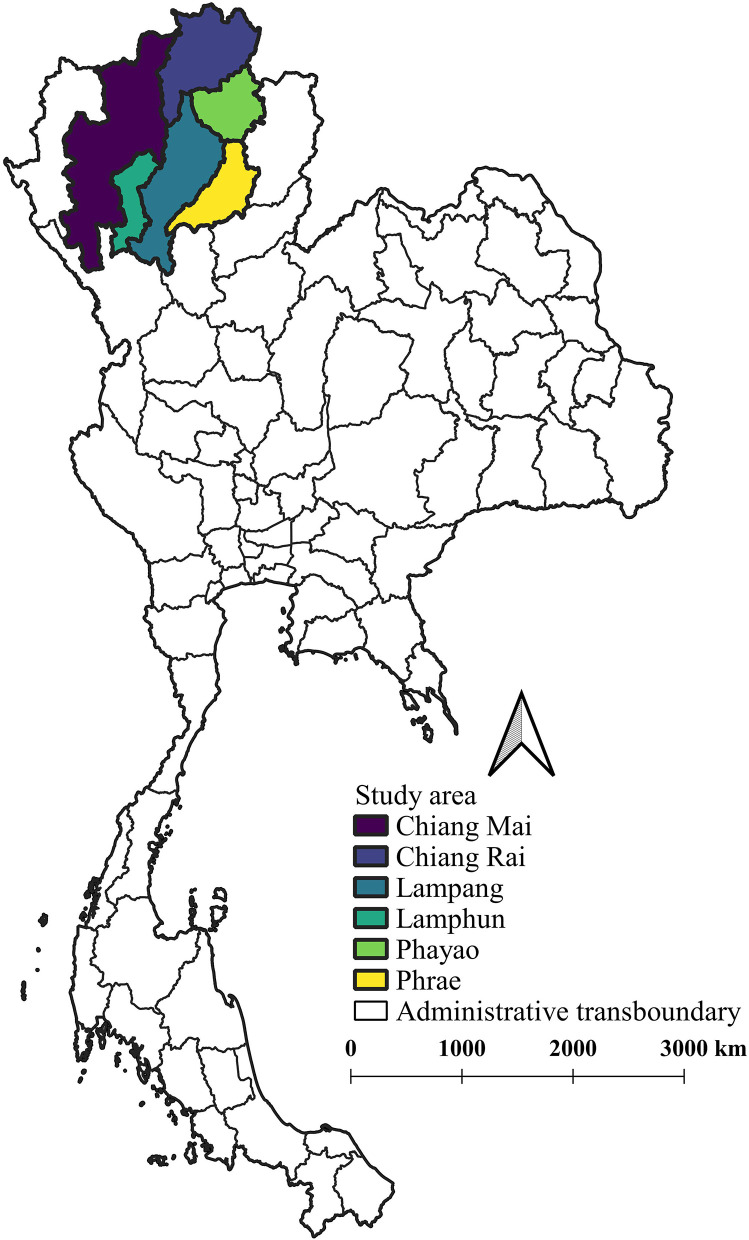
A map of the provinces in northern Thailand where milk production data were collected. The map was created using QGIS (version 2.18.28), QGIS Geographic Information System, Open Source Geospatial Foundation Project, all content is licensed under Creative Commons Attribution ShareAlike 3.0 license (CC BY-SA), available at (http://qgis.osgeo.org).

The milk production data from all dairy cooperatives and private milk collection centers in all northern provinces (*n* = 6) were collected monthly and then verified by livestock authorities from the Department of Livestock Development. Furthermore, data on milk production were summarized in order to represent the monthly milk production of the northern region's dairy sector. The provinces in northern Thailand from which milk production data were collected are shown in Figure 1.

We have two different approaches for data management and data analysis based on the objectives of the study. The first approach was to develop forecast models and predict the milk production using the entire dataset (full dataset). The second approach was to build forecast models and then compared forecast values with the actual data value by holding the last 12 months of the data as a validation data (validation dataset) and developing models from the remaining data (training dataset). Hence, milk production data from the full dataset (January 2016–December 2020) was divided into training (January 2016–December 2019) and validation datasets (January 2020–December 2020). The training dataset was used for model development. Furthermore, the final models obtained from this training dataset were evaluated for their performance using the validation dataset. By this process, we could compare the model performances among models developed. Similar to the process using the training dataset, the full dataset was used to develop models to forecast milk production for the period of January 2021–December 2022. Notably, the advantage of using the full dataset was its update. If the training dataset was used to forecast, the models would be trained only for 2016–2019 data, which might affect the forecast of milk production for 2021–2022 due to the long-term forecast effect that may contribute to a decrease in forecast accuracy. Since we used the full dataset to developed the models, the forecast for the period of 2021–2022 is less likely to be affected by this constraint, providing the relevant users and policy makers a more accurate prediction.

### Statistical Analysis

Data were analyzed using R statistical software version 4.0.4 and relevant packages (46). Raw milk production data were converted to time-series data using functions from the “xts” package and then decomposed with functions from “TSstudio” packages. To develop forecast models, several functions from a “forecast” package were used.

### Time-Series Data Decomposition

The time-series milk production data were decomposed into trend, season, and error to facilitate separate examination for each of them.

Additive time-series data consisted of trend, season, and irregular (error) components, and the model is given as follows (26):


(1) $$\begin{array}{l} \begin{array}{c} y_{t}=T_{t}+S_{t}+I_{t} \end{array} \end{array}$$

where *y*_*t*_is the milk production value at time *t*, *T*_*t*_ is the trend-cycle component at time *t*, *S*_*t*_ is the seasonal component at time *t*, and *I*_*t*_ is the irregular (remainder) component at time *t*.

### Model Development

For the pre-modeling steps, we examined the milk production data by testing the following assumptions including (i) the stationary of time-series data using Augmented Dickey-Fuller Test, (ii) the heteroscedasticity in a time series using an autoregressive conditionally heteroscedastic test (ARCH test), and (iii) autocorrelation of time-series data using autocorrelation function (ACF) and partial autocorrelation function (PACF) plots.

### SARIMA Model

The SARIMA model (26) is expressed as


(2) $$\begin{array}{l} \begin{array}{c} \phi_{p}\left(B\right)\Phi_{P}\left(B^{s}\right){\left(1-B\right)}^{d}{\left(1-B^{s}\right)}^{D}y_{t}=\theta_{q}\left(B\right)\Theta_{Q}\left(B^{s}\right)\varepsilon_{t} \end{array} \end{array}$$

where ϕ and θ are parameters of the autoregressive and moving average, whereas Φ and Θ are parameters of the seasonal autoregressive and seasonal moving average, respectively. Meanwhile, *p, d*, and *q* are the order of autoregressive, degree of differencing, and order of moving average, respectively. Additionally, *P, D*, and *Q* are the orders of the seasonal autoregressive, degree of seasonal differencing, and order of seasonal moving average, respectively. *S* is the seasonal length, *y*_*t*_ is predicted variable, and ε_*t*_ is a random error at time *t*.

The function *auto.arima* from the “forecast” package (47) was applied to the milk production data to determine the best fitting ARIMA model. The *auto.arima* algorithm performed various steps to select the model (26). In brief, the steps were given as follows—(i) if the data were non-stationary, the algorithm would determine the number of differences in the range of 0 ≤ d ≤ 2 using repeated Kwiatkowski-Phillips-Schmidt tests until they became stationary, (ii) the values of *p* and *q* were chosen by minimizing the corrected Akaike Information Criterion (AICc) after differencing the data *d* times, (iii) the algorithm searched for a combination of *p* and *q* using a stepwise approach, and (iv) the algorithm repeated the search to evaluate AICc, and variations could be found until they reach the lowest AICc. The model with the lowest AICc was defined as the final model and used for further predictions (26, 47).

### ETS Model

For ETS methods, the forecast was made considering a weighted average of past observation. The latest observation is given exponentially more weight than earlier observations (48). The state-space model of the ETS was defined as ETS (., ., .) for (Error, Trend, Seasonal). The possibilities for each component are Error = (A, M), Trend = (N, A, A_d_), and Seasonal = (N, A, M). The letters A, M, N, and A_d_ refer to additive, multiplicative, none, and additive damped, respectively (29). For instance, the ETS (A, A, M) was the method with the additive trend, multiplicative seasonality and additive errors (25, 26). By using the state-space structure, the optimal exponential smoothing model could be determined. According to the model building algorithm from the “forecast” package, the *ets* function was utilized to determine the final ETS model, defined as the model with the lowest AICc value among models developed (26).

### Hybrid Model

Several functions from the “forecast” and “forecastHybrid” packages (26, 49) were employed to develop a hybrid model of SARIMA and ETS (SARIMA-ETS). Since functions from “forecastHybrid” R package developed by Shaub and Ellis offer the automated procedure to obtain the best fitting model (27); thus, the *hybridModel* function from this package was employed to develop the SARIMA-ETS model. According to this procedure, forecast values generated from *auto.arim* and *ets* functions were combined equally to develop the hybrid model. The automatic methodology from the *hybridModel* function provided results obtained by optimizing the prediction features of the model-based minimizing error (27). The detail of these procedures was previously described (49, 50).

### Model Assumption Diagnostics

Residuals from all final models were tested for model assumptions. The Ljung–Box Q test would be used to diagnose if the residual error sequence from the final model was a white-noise sequence (51). In addition, residuals from the final model were plotted to examine autocorrelation and partial autocorrelation functions (26). All of these methods for checking residuals were performed using a function *checkresiduals* from the “forecast” package, which produced a time plot, ACF plot, and histogram of the residuals (with an overlaid normal distribution for comparison) and conducted a Ljung-Box test (26).

### Model Performance and Forecast

With the validation dataset, error matrices that were commonly used including mean absolute error (MAE), root mean square error (RMSE), and mean absolute percent error (MAPE) were calculated to determine forecast performances among the developed models (26, 52). It is generally accepted that the lower the measure error matric values, the better the method (33).

As described previously, the forecasts of milk production were estimated from the models developed from training and full datasets. Thus, we forecasted milk production for the periods of January-December 2020 and January 2021–December 2022 using training and full datasets, respectively. The performance of forecast models was determined by comparing the milk production forecast values from forecast models applied to the training dataset with actual milk production values from the validation dataset. In addition, forecast values for milk production for the period of January 2021-December 2022 were computed using forecast models applied to the full dataset.

## Results

### Decomposition

**Figure 2:**
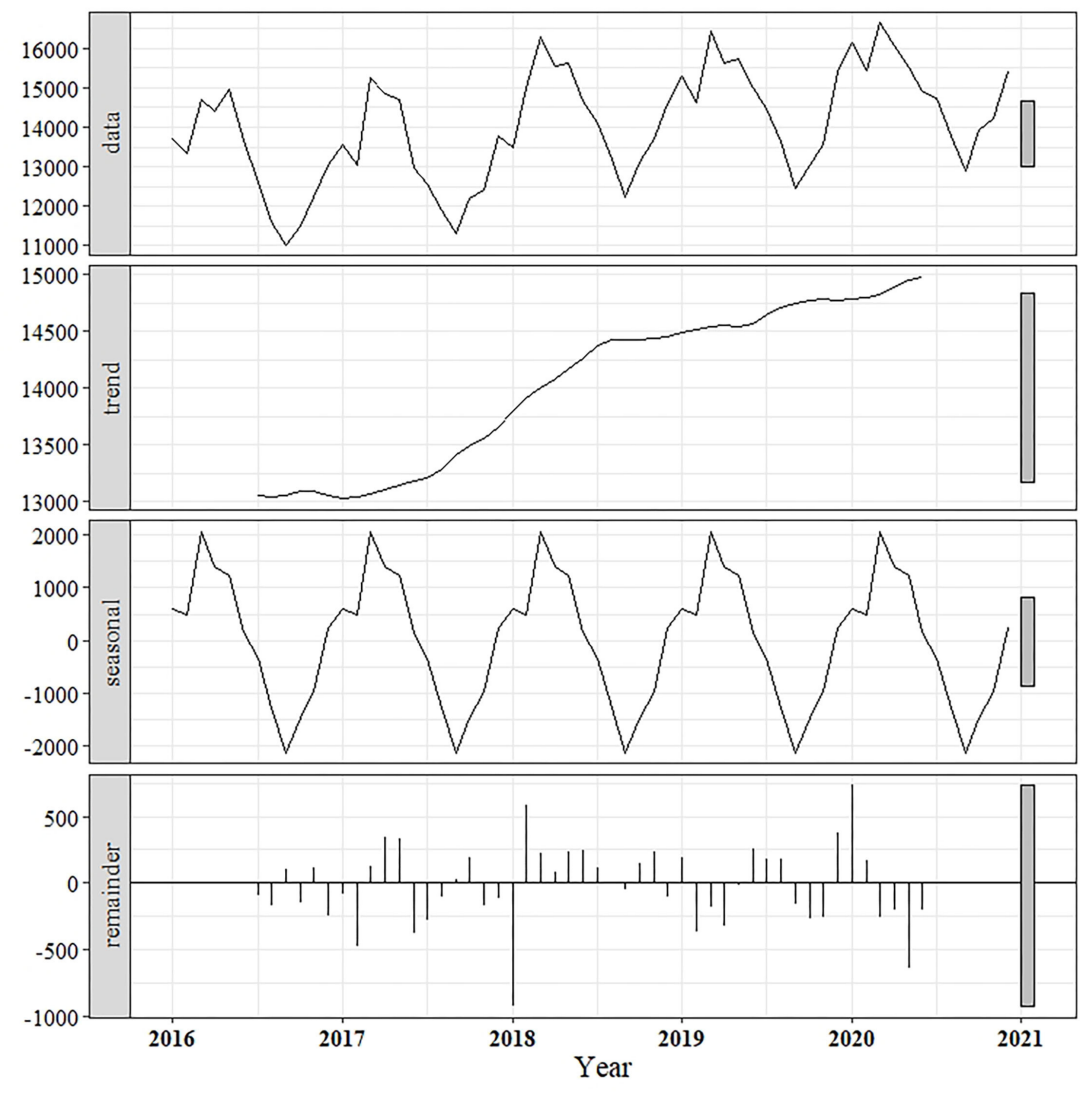
Decomposition of time-series milk production data (January 2016–December 2020) into trend, seasonal, and error (remainder) components.

Overall, the actual monthly milk production showed an increasing trend with the existence of seasonal fluctuation (Figure 2). Upon decomposition of the actual data into trends, it was revealed that a consistently increasing trend in milk production could be observed over the period 2017–2018, and the milk production was gradually raised from 2019 to 2020. When the data was decomposed into a seasonal component, a seasonal pattern was clearly shown, with the predominant peak in milk production occurring between March and May every year (Figure 2).

### Models

Based on the ARIMA approach, the SARIMA (1,0,0) (1,1,0)_12_ was defined as the final model. This model is interpreted as follows: the number of lag observations included in the model or lag order is equal to one (*p* = 1), the degree of differences is equal to zero (*d* = 0), the order of moving average is equal to zero (*q* = 0), the last seasonally offset observation is used in the model (*P* =1), seasonal differences are equal to one (*D* = 1) and moving average order in seasonality is equal to zero (*Q* =0), and yearly seasonal is set (*m* = 12). For the ETS approach, the ETS (M, A, A) was selected as the final model which could be interpreted as the model with an additive trend (A), additive seasonality (A), and multiplicative errors (M). Moreover, the hybrid model of SARIMA-ETS was determined as well.

Results according to model assumption testing including ACF plots and the Ljung–Box test are shown in the Supplementary Material S1.

### Model Performance

**Figure 3:**
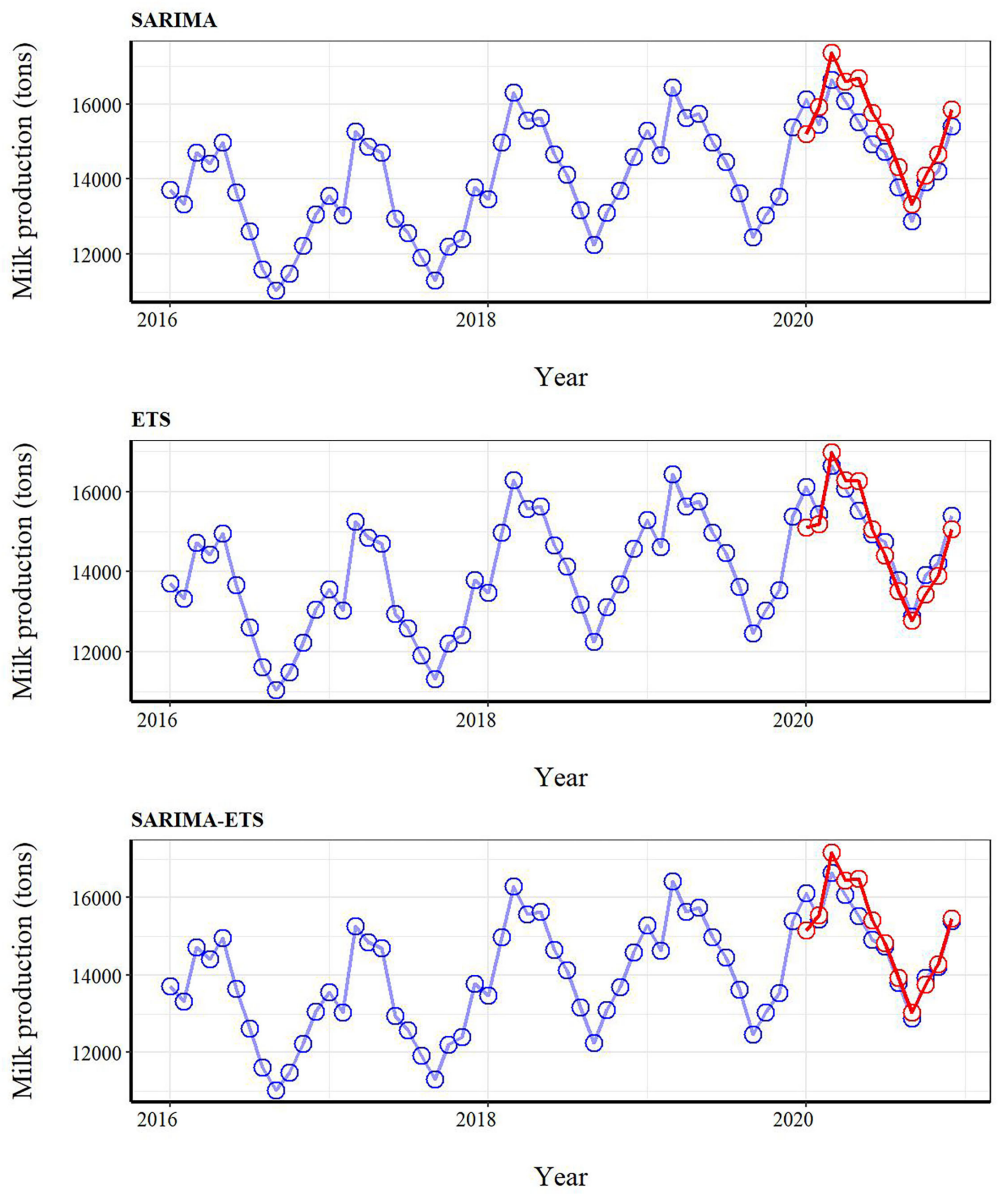
The actual milk production (blue circles) from the full dataset (January 2016–December 2020) and forecast milk production values (red circles) for January-December 2020 derived from seasonal autoregressive integrated moving average (SARIMA), error trend seasonality (ETS), and SARIMA-ETS hybrid models applied to the training dataset. The performance of time series models was measured by comparing forecasted and milk production values from January to December 2020.

The SARIMA-ETS model performed better than other models as it had the lowest MAE, RMSE, and MAPE values (Table 1). This finding implied that the hybrid model approach had a better forecast accuracy compared with the single model approach. The actual and predicted values for milk production based on the validation dataset are shown in Figure 3. It was demonstrated that several SARIMA-ETS forecast values were remarkedly closer to the actual values of milk production than those from other models. Notably, for the last six months of the testing dataset, the hybrid model predicted milk production with a high degree of accuracy, whereas the ETS appeared to perform well in prediction over the course of a year.

**Table 1:** Error matrices for time series and hybrid models applied to the validation dataset.

Model^*^	**MAE^a^**	**RMSE^b^**	**MAPE^c^**
SARIMA	600.11	652.64	3.96
ETS	382.60	458.40	2.52
SARIMA-ETS	342.36	467.71	2.21

* *SARIMA, seasonal autoregressive integrated moving average; ETS, error trend seasonality; SARIMA-ETS, hybrid model of SARIMA and ETS*.

a *MAE, mean absolute error*.

b *RMSE, root mean square error*.

c *MAPE, mean absolute percent error*.

### Milk Production Forecast

**Figure 4:**
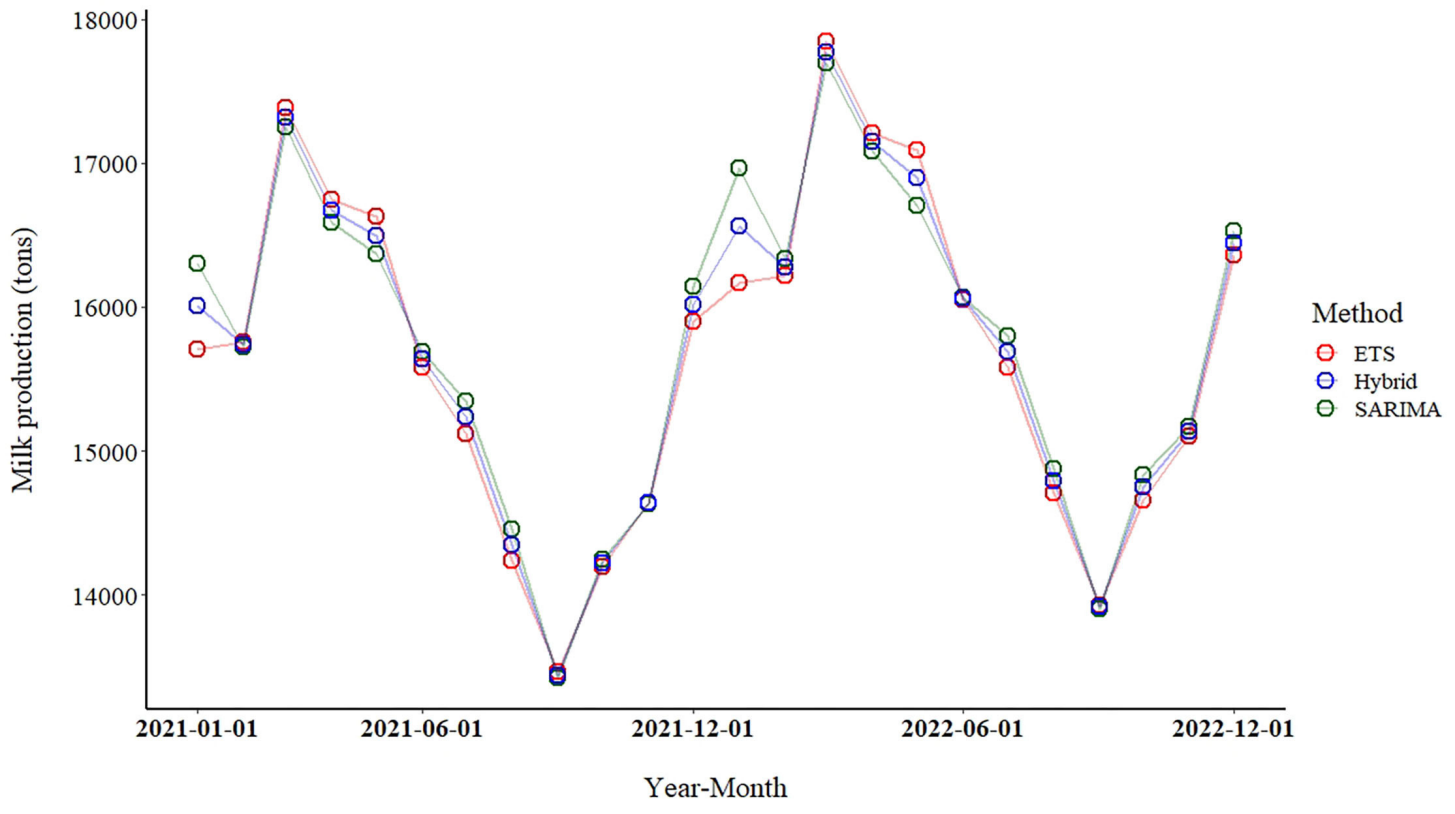
Forecasts of milk production (January 2021–December 2022) using the seasonal autoregressive integrated moving average (SARIMA), error trend seasonality (ETS), and SARIMA-ETS hybrid models applied to the dataset of January 2016–December 2020. Green, red and blue circles represent to the forecast values from SARIMA, ETS and SARIMA-ETS, respectively.

According to the ETS, SARIMA-ETS and SARIMA models, milk production was expected to increase by 3.2, 3.4 and 3.6% per year between 2021 and 2022, respectively. The forecast values of milk production from all model are illustrated in Figure 4 and presented in the Supplementary Material S2. Results from the best performance model (SARIMA-ETS) highlighted an increasing trend of milk production with fluctuation due to the seasonality. The SARIMA-ETS hybrid model delivered forecast values that were all in the middle of forecast values from SARIMA and ETS methods. Notably, the SARIMA provided the highest forecast values for 14 of the 24 months of the prediction.

## Discussion

Oversupply of milk is a matter of serious concern for Thailand's dairy industry (7, 53). Therefore, establishing accurate forecast models for milk production becomes the cornerstone of efficient planning and management. In this study, we developed and evaluated forecast models using various time-series statistical methods to predict milk production. The novelty of this study lies in the fact that milk production data were firstly analyzed using the hybrid forecasting model.

It is generally accepted that there is no one-size-fits-all forecast method for various data types and each one comes with its own merits (27, 54). Thus, numerous models based on several forecast methods to predict milk production were developed. The findings from this study demonstrated that the ETS model performed better than the SARIMA model. This finding can be explained by the fact that each method has a different capacity for dealing with the data. Although both methods have a great capability to analyze data with seasonal patterns, the SARIMA has difficulties in detecting and considering the non-linear pattern of the data (15, 55) which may exist in our data. On the other hand, the state-space methods of the ETS can capture both linear and non-linear patterns of the time-series data (32, 56). Hence, it provided a higher forecast accuracy. Our study also highlighted that the hybrid model outperformed the single model, which has been supported by numerous previous studies (33, 52, 57, 58). In this study, the SARIMA tend to overestimate the milk productions, but the ETS appeared to underestimate them. The most accurate forecast model was obtained from the SARIMA-ETS hybrid model. The better forecast performance of the hybrid model over the single model observed in this study was likely attributed to its capability to capture irregular fluctuations, seasonality patterns, and other data behaviors (27, 52) because the hybrid model combined strengths of the single model. While the SARIMA performed well in dealing with the autocorrelation in data, the ETS has a great capability to deal with the trend and irregular patterns (59). Indeed, the hybrid model combines the strengths of each model that can work on its own expertise. In addition, the hybrid model can make the model work together to overcome other weaknesses (60). Thus, the hybrid model was expected to deliver more accurate predictions compared to the single model (61) as observed in the present and numerous previous studies (11, 55, 57, 60).

The present study provided the estimation of future milk production from the northern region, which policymakers could combine with other milk production data from other regions to establish a plan for managing the overall milk production in Thailand. In this study, we identified the major challenges regarding the future milk production in the north including the increasing trend of milk production for the period of 2021–2022 and the fluctuation in the production due to seasonality (Figure 4). The upward trend of milk production poses a challenge to authorities and stakeholders along the milk supply chain to formulate a strategic plan to organize future milk production. Identifying new potential target consumers or expanding milk markets may be a part of the plan. Moreover, our prediction results suggested that the milk production during some months was relatively high compared with other months due to the seasonality. Thus, we suggested that milk marketing should be planned to manage this fluctuation.

Regarding previous reports from other countries such as Bangladesh, India, and Pakistan, milk production forecasts are limited to ARIMA models (43, 62, 63). Accordingly, the lack of the application of other classical and hybrid models to fit the data may result in some knowledge gaps in the pursuit of a better forecasting strategy for this type of data. To the best of our knowledge, this was the first study to forecast milk production utilizing ETS and hybrid models. However, it's worth noting that the best-fitting model in this study may not be well-suited for all milk production data. Thus, for future studies, we suggest applying several appropriate forecast models to a particular milk production dataset and then determine the best-suited model. For a follow-up study in Thailand, we recommend establishing forecast models for milk production using nationwide data. Herein, it was the first study for our ongoing project, and the method selected to be utilized was based on the nature of milk production data in Thailand, which obviously has a seasonal pattern; however, an application of other advanced forecast models is warranted for follow-up studies.

In summary, we applied time-series statistical methods including SARIMA, ETS, and SARIMA-ETS hybrid models to analyze and forecast milk production. Our findings indicated a continuously increasing trend of milk production in the northern region of Thailand from 2016 to 2020. The most preferred model was the SARIMA-ETS model yielded the lowest values of error matrices. Our results have provided policymakers and stakeholders with useful information for developing an effective strategic plan for managing milk volumes in the coming years. This approach is not limited to the data used in this study; it can be applied to an updated dataset to produce ongoing forecasts.

## Data Availability Statement

The original contributions presented in the study are included in the article/Supplementary Materials, further inquiries can be directed to the corresponding author.

## Author Contributions

VP, KJ, and CS designed the study, analyzed the data, and wrote the main manuscript text. VP, CS, and KK collected, managed, and visualized the data. VP provided oversight for the study and draft the manuscript. VP, KJ, KK, and CS contributed to the interpretation of the result and reviewed the manuscript. All authors contributed to the article and approved the submitted version.

## Funding

This work was funded by Chiang Mai University (Grant numbers: R000026522 and R000026062). The funder had no role in the study design, data analysis, decision to publish, or manuscript preparation.

## Conflict of Interest

The authors declare that the research was conducted in the absence of any commercial or financial relationships that could be construed as a potential conflict of interest.

## Publisher's Note

All claims expressed in this article are solely those of the authors and do not necessarily represent those of their affiliated organizations, or those of the publisher, the editors and the reviewers. Any product that may be evaluated in this article, or claim that may be made by its manufacturer, is not guaranteed or endorsed by the publisher.

## References

[1] The Department of Trade Negotiations. Milk and Milk Product. (2020). Available online at: https://api.dtn.go.th/files/v3/5e87153fef414065c970d1c5/download (accessed January 12, 2021).

[2] The Department of Livestock Development. Statistics for Livestocks and Farmers. (2021). Available online at: http://ict.dld.go.th/webnew/index.php/th/service-ict/report/352-report-thailand-livestock/reportservey2564/1530-2564-monthly (accessed January 18, 2021).

[3] The Department of Livestock Development. Strategic Plan for Dairy in the Northern of Thailand. (2020). Available online at: http://region5.dld.go.th/webnew/index.php/th/organization-menu-2/strategic-menu/1211-2011-03-01-13-38-36 (accessed January 18, 2021).

[4] The Department of Livestock Development. Strategic Plan for Dairy in the Northern of Thailand. (2020). Available online at: http://region5.dld.go.th/webnew/images/stories/2563/yut/yutdairycattle.pdf (accessed January 18, 2021).

[5] Jitmun T Kuwornu JK Datta A Anal AK. Farmers' perceptions of milk-collecting centres in Thailand's dairy industry. Dev Pract. (2019) 29:424–36. 10.1080/09614524.2019.1568394

[6] Aiumlamai S Kreausukon K Wongnen N. In Planning Dairy Development Programs in Asia. In: Proceedings of a Symposium held at 15th AAAP Congress. Bangkok (2012). Available online at: http://cdn.aphca.org/dmdocuments/Proceedings_dairy.pdf (accessed January 18, 2021).

[7] The Department of Cooperative Promotion. Thai Milk Board Meeting. (2020). Available online at: https://www.cpd.go.th/cpdth2560/component/k2/newcpd_08dec2563_1 (accessed January 18, 2021).

[8] The Department of Livestock Development. Strategic Plan for Dairy and Dairy Product. (2020). Available online at: https://biotech.dld.go.th/webnew/Data/School-Milk/09082563.pdf (accessed January 18, 2021).

[9] Yothasamut J Camfield L Pfeil M. Practices and values regarding milk consumption among pre-schoolers in Bangkok. Int J Qual Stud Health Well-being. (2018) 13:1461515. 10.1080/17482631.2018.1461515 29667877 PMC5906937

[10] Anyday Guide. National Dairy Day in Thailand. (2021). Available online at: https://anydayguide.com/calendar/3826 (accessed January 18, 2021).

[11] Hajirahimi Z Khashei M. Hybrid structures in time series modeling and forecasting: a review. Eng Appl Artif Intell. (2019) 86:83–106. 10.1016/j.engappai.2019.08.018

[12] Sezer OB Gudelek MU Ozbayoglu AM. Financial time series forecasting with deep learning: a systematic literature review: 2005–2019. Appl Soft Comput. (2020) 90:106181. 10.1016/j.asoc.2020.106181

[13] Goldberg MS Burnett RT Stieb D. A review of time-series studies used to evaluate the short-term effects of air pollution on human health. Rev Environ health. (2003) 8:269–303. 10.1515/REVEH.2003.18.4.269 15025190

[14] Chan HK Xu S Qi X. A comparison of time series methods for forecasting container throughput. Int J Logistics Res Appl. (2019) 22:294–303. 10.1080/13675567.2018.1525342

[15] Panigrahi S Behera HS. A hybrid ETS–ANN model for time series forecasting. Eng Appl Artif Intell. (2017) 66:49–59. 10.1016/j.engappai.2017.07.007

[16] Dong Y Li S Gong X editors. Time series analysis: an application of arima model in stock price forecasting. In: *International Conference on Innovations in Economic Management and Social Science (IEMSS 2017)*. Atlantis Press. (2017). p. 149–74. 10.2991/iemss-17.2017.140

[17] Yildiran CU Fettahoglu A. Forecasting USDTRY rate by ARIMA method. Cogent Econ Financ. (2017) 5:1. 10.1080/23322039.2017.1335968

[18] Liu L Luan R Yin F Zhu X Lü Q. Predicting the incidence of hand, foot and mouth disease in Sichuan province, China using the ARIMA model. Epidemiol Infect. (2016) 144:144–51. 10.1017/S0950268815001144 26027606 PMC9507307

[19] Wang YW Shen ZZ Jiang Y. Comparison of ARIMA and GM (1, 1) models for prediction of hepatitis B in China. PLoS ONE. (2018) 13:e0201987. 10.1371/journal.pone.0201987 30180159 PMC6122800

[20] Alzahrani SI Aljamaan IA Al-Fakih EA. Forecasting the spread of the COVID-19 pandemic in Saudi Arabia using ARIMA prediction model under current public health interventions. J Infect Public Health. (2020) 13:914–9. 10.1016/j.jiph.2020.06.001 32546438 PMC7837129

[21] Ward M Iglesias R Brookes VJ. Autoregressive models applied to time-series data in veterinary science. Front Vet Sci. (2020) 7:604. 10.3389/fvets.2020.00604 33094106 PMC7527444

[22] Lee HS Her M Levine M Moore GE. Time series analysis of human and bovine brucellosis in South Korea from 2005 to 2010. Prev Vet Med. (2013) 110:190–7. 10.1016/j.prevetmed.2012.12.003 23276400

[23] Nguyen XH. Combining statistical machine learning models with ARIMA for water level forecasting: the case of the Red river. Adv Water Resour. (2020) 142:103656. 10.1016/j.advwatres.2020.103656

[24] Praveen B Sharma P. Climate variability and its impacts on agriculture production and future prediction using autoregressive integrated moving average method (ARIMA). J Public Affairs. (2020) 20:e2016. 10.1002/pa.201625855820

[25] Heydari M Ghadim HB Rashidi M Noori M. Application of holt-winters time series models for predicting climatic parameters (case study: Robat Garah-Bil Station, Iran). Polish J Environ Stud. (2019) 29:617–27. 10.15244/pjoes/100496

[26] Hyndman RJ Athanasopoulos G. Forecasting: Principles and Practice, 2nd Edn. Melbourne, VIC: OTexts (2018). Available online at: OTexts.com/fpp2 (accessed January 18, 2021).

[27] Gjika E Aurora F Arbesa K. A study on the efficiency of hybrid models in forecasting precipitations and water inflow albania case study. Adv Sci Technol Eng Syst J. (2019) 4:302–10. 10.25046/aj040129

[28] Shih H Rajendran S. Comparison of time series methods and machine learning algorithms for forecasting Taiwan Blood Services Foundation's blood supply. J Healthc Eng. (2019) 2019:6123745. 10.1155/2019/6123745 31636879 PMC6766103

[29] Hyndman R Koehler AB Ord JK Snyder RD. Forecasting With Exponential Smoothing: The State Space Approach. Berlin: Springer (2008). p. 372. 10.1007/978-3-540-71918-2

[30] Wang Y Xu C Ren J Wu W Zhao X Chao L. Secular seasonality and trend forecasting of tuberculosis incidence rate in China using the advanced error-trend-seasonal framework. Infect Drug Resistance. (2020) 13:733–47. 10.2147/IDR.S238225 32184635 PMC7062399

[31] Wang K Deng C Li J Zhang Y Li X Wu M. Hybrid methodology for tuberculosis incidence time-series forecasting based on ARIMA and a NAR neural network. Epidemiol Infect. (2017) 145:1118–29. 10.1017/S0950268816003216 28115032 PMC9507834

[32] Andellini M Bassanelli E Faggiano F Esposito MT Marino S Ritrovato M editors. Forecasting hospital performances using a hybrid ETS-ARIMA algorithm. In: International Workshop on Metrology for Industry 40 & IoT. Rome (2021). Available online at: https://www.metroind40iot.org/MetroInd2021_FinalProgram_v3.pdf (accessed January 18, 2021). 10.1109/MetroInd4.0IoT51437.2021.9488500

[33] Azeez A Obaromi D Odeyemi A Ndege J Muntabayi R. Seasonality and trend forecasting of tuberculosis prevalence data in Eastern Cape, South Africa, using a hybrid model. Int J Environ Res Public Health. (2016) 13:757. 10.3390/ijerph13080757 27472353 PMC4997443

[34] Wang Y Xu C Zhang S Wang Z Yang L Zhu Y. Temporal trends analysis of tuberculosis morbidity in mainland China from 1997 to 2025 using a new SARIMA-NARNNX hybrid model. BMJ Open. (2019) 9:e024409. 10.1136/bmjopen-2018-024409 31371283 PMC6678063

[35] Faruk DÖ. A hybrid neural network and ARIMA model for water quality time series prediction. Eng Appl Artif Intell. (2010) 23:586–94. 10.1016/j.engappai.2009.09.015

[36] Uddin M Akter A Khaleduzzaman A Sultana M. Forecasting milk production in Bangladesh toward achieving self-sufficiency. Livestock Res Rural Dev. (2020) 32:2020. Retrieved from: http://www.lrrd.org/lrrd32/5/moham32081.html.

[37] Mishra P Matuka A Abotaleb MSA Weerasinghe W Karakaya K Das S. Modeling and forecasting of milk production in the SAARC countries and China. Model Earth Syst Environ. (2021) 7:1–13. 10.1007/s40808-021-01138-z

[38] Taye BA Alene AA Nega AK Yirsaw BG. Time series analysis of cow milk production at Andassa dairy farm, west Gojam zone, Amhara region, Ethiopia. Model Earth Syst Environ. (2021) 7:181–9. 10.1007/s40808-020-00946-z

[39] Deshmukh SS Paramasivam R. Forecasting of milk production in India with ARIMA and VAR time series models. Asian J Dairy Food Res. (2016) 35:17–22. 10.18805/ajdfr.v35i1.9246

[40] Thissen U Van Brakel R De Weijer A Melssen W Buydens L. Using support vector machines for time series prediction. Chemometr Intell Lab Syst. (2003) 69:35–49. 10.1016/S0169-7439(03)00111-424808276

[41] do Nascimento Camelo H Lucio PS Junior JBVL de Carvalho PCM. A hybrid model based on time series models and neural network for forecasting wind speed in the Brazilian northeast region. Sustain Energy Technol Assess. (2018) 28:65–72. 10.1016/j.seta.2018.06.009

[42] Punyapornwithaya V Klaharn K Sansamur C Kitpipit W. Trend and seasonality analysis of milk production from dairy cooperatives in Chiang Mai. Vet Integr Sci. (2021) 19:101–10. Retrieved from: https://he02.tci-thaijo.org/index.php/vis/article/view/246619.

[43] Ahmed F Shah H Raza I Saboor A. Forecasting milk production in Pakistan. Pak J Agric Res. (2011) 24:82–5. Available online at: http://pjar.org.pk/Issues/Vol24_2011No1_4/Vol24No1_4Page82.pdf. (accessed January 12, 2021).

[44] O'Connell A McParland S Ruegg P O'Brien B Gleeson D. Seasonal trends in milk quality in Ireland between 2007 and 2011. J Dairy Sci. (2015) 98:3778–90. 10.3168/jds.2014-9001 25828653

[45] Oltenacu P Smith T Kaiser H. Factors associated with seasonality of milk production in New York state. J Dairy Sci. (1989) 72:1072–9. 10.3168/jds.S0022-0302(89)79205-5

[46] R Core Team. Version 4.1.2. R: A Language and Environment for Statistical Computing. R Foundation for Statistical Computing. Vienna: R Foundation for Statistical Computing (2021).

[47] Hyndman RJ Athanasopoulos G Bergmeir C Caceres G Chhay L O'Hara-Wild M. Package ‘Forecast'. Available online at: https://cranr-projectorg/web/packages/forecast/forecastpdf (accessed January 18, 2021).

[48] Hidayatulah H Parasian S. Comparison of forecasting accuracy rate of exponential smoothing method on admission of new students. J Critic Rev. (2020) 2:268–74. 10.31838/jcr.07.02.50

[49] Shaub D. Fast and accurate yearly time series forecasting with forecast combinations. Int J Forecast. (2020) 36:116–20. 10.1016/j.ijforecast.2019.03.032

[50] Shaub D Ellis P. Forecast Hybrid: Convenient Functions for Ensemble Time Series Forecasts. (2020). Available online at: https://github.com/ellisp/forecastHybrid (accessed January 12, 2021).

[51] Liu H Li C Shao Y Zhang X Zhai Z Wang X. Forecast of the trend in incidence of acute hemorrhagic conjunctivitis in China from 2011–2019 using the Seasonal Autoregressive Integrated Moving Average (SARIMA) and Exponential Smoothing (ETS) models. J Infect Public Health. (2020) 13:287–94. 10.1016/j.jiph.2019.12.008 31953020

[52] Perone G. Comparison of ARIMA, ETS, NNAR, TBATS and hybrid models to forecast the second wave of COVID-19 hospitalizations in Italy. Eur J Health Econ. (2021) 4:1–24. 10.1007/s10198-021-01347-4 34347175 PMC8332000

[53] The Department of Livestock Development. Strategic Plan for Dairy and Dairy Product. (2020). Available online at: https://www.cpd.go.th/cpdth2560/component/k2/newcpd_08dec2563_1 (accessed January 27, 2021).

[54] Athiyarath S Paul M Krishnaswamy S. A comparative study and analysis of time series forecasting techniques. SN Comput Sci. (2020) 1:1–7. 10.1007/s42979-020-00180-5

[55] Wang L Zou H Su J Li L Chaudhry S. An ARIMA-ANN hybrid model for time series forecasting. Syst Res Behav Sci. (2013) 30:244–59. 10.1002/sres.217925855820

[56] Yusof F Kane I. Modelling monthly rainfall time series using ETS state space and SARIMA models. Int J Curr Res. (2012) 4:195–200. Available online at: https://www.journalcra.com/article/modeling-monthly-rainfall-time-series-using-ets-state-space-and-sarima-models (accessed January 14, 2021).

[57] Wang Y Xu C Li Y Wu W Gui L Ren J. An advanced data-driven hybrid model of SARIMA-NNNAR for tuberculosis incidence time series forecasting in Qinghai Province, China. Infect Drug Resist. (2020) 13:867–80. 10.2147/IDR.S232854 32273731 PMC7102880

[58] Mohammed SH Ahmed MM Al-Mousawi AM Azeez A. Seasonal behavior and forecasting trends of tuberculosis incidence in Holy Kerbala, Iraq. Int J Mycobacteriol. (2018) 7:361–7. 10.4103/ijmy.ijmy_109_18 30531036

[59] Ramos P Santos N Rebelo R. Performance of state space and ARIMA models for consumer retail sales forecasting. Robot Comput Integr Manufact. (2015) 34:151–63. 10.1016/j.rcim.2014.12.015

[60] Khandelwal I Adhikari R Verma G. Time series forecasting using hybrid ARIMA and ANN models based on DWT decomposition. Procedia Comput Sci. (2015) 48:173–9. 10.1016/j.procs.2015.04.167

[61] Dave E Leonardo A Jeanice M Hanafiah N. Forecasting Indonesia exports using a hybrid model ARIMA-LSTM. Procedia Comput Sci. (2021) 179:480–7. 10.1016/j.procs.2021.01.031

[62] Paul RK Alam W Paul A. Prospects of livestock and dairy production in India under time series framework. Indian J Anim Sci. (2014) 84:462–6. Available online at: http://krishi.icar.gov.in/PDF/ICAR_Data_Use_Licence.pdf (accessed January 12, 2021).

[63] Suresh K Priya SK. Forecasting sugarcane yield of Tamilnadu using ARIMA models. Sugar Tech. (2011) 13:23–6. 10.1007/s12355-011-0071-7

