# Supplementary material for: Forecasting of Milk Production in Northern Thailand Using Seasonal Autoregressive Integrated Moving Average, Error Trend Seasonality, and Hybrid Models
Source: Front Vet Sci. 2021 Nov 30;8:775114. doi: 10.3389/fvets.2021.775114 (PMC8669476; doi:10.3389/fvets.2021.775114)
Supplement: Supplementary file 1 [file Data_Sheet_1.docx]

**Supplementary S1**

Model assumption diagnostics were performed by checking residuals using a function *checkresiduals* form R “forecast” and “ggfortify” packages, which produced a time plot, ACF plot and histogram of the residuals and conducted a Ljung-Box test.

1. Seasonal Autoregressive Integrated Moving Average (SARIMA) model


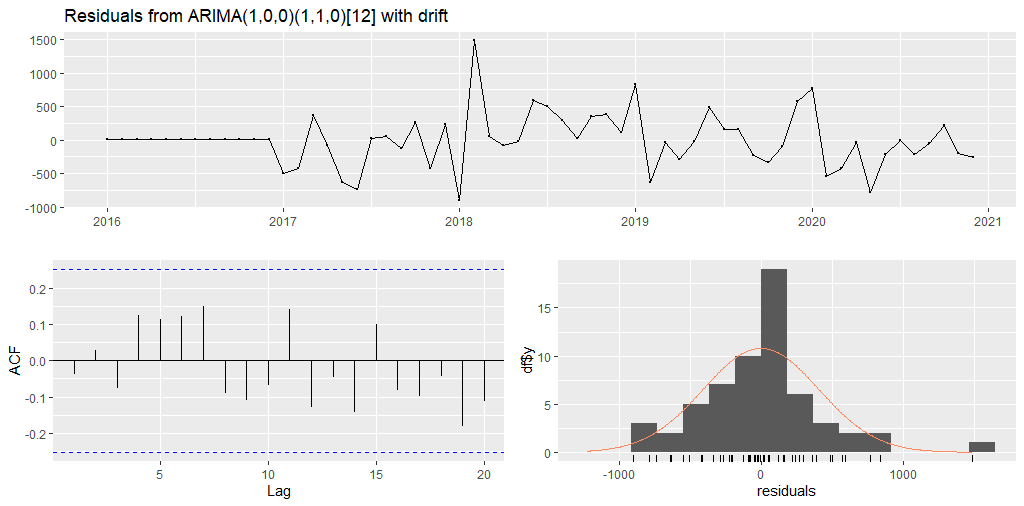


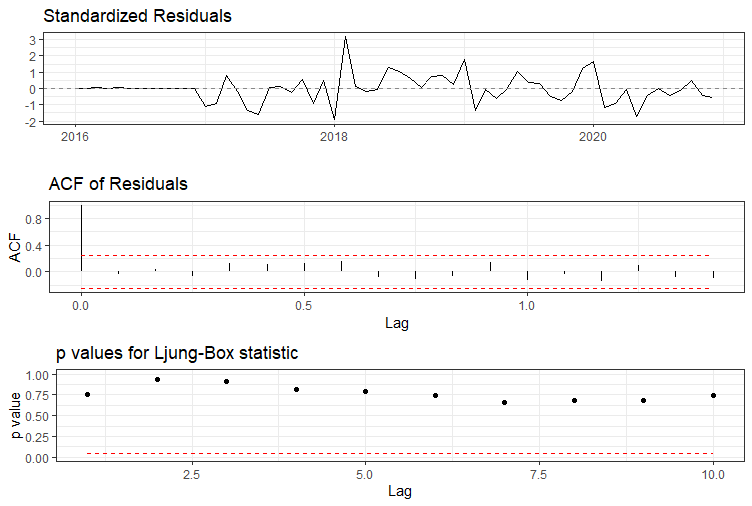


1. Error, Trend and Seasonality (ETS) model


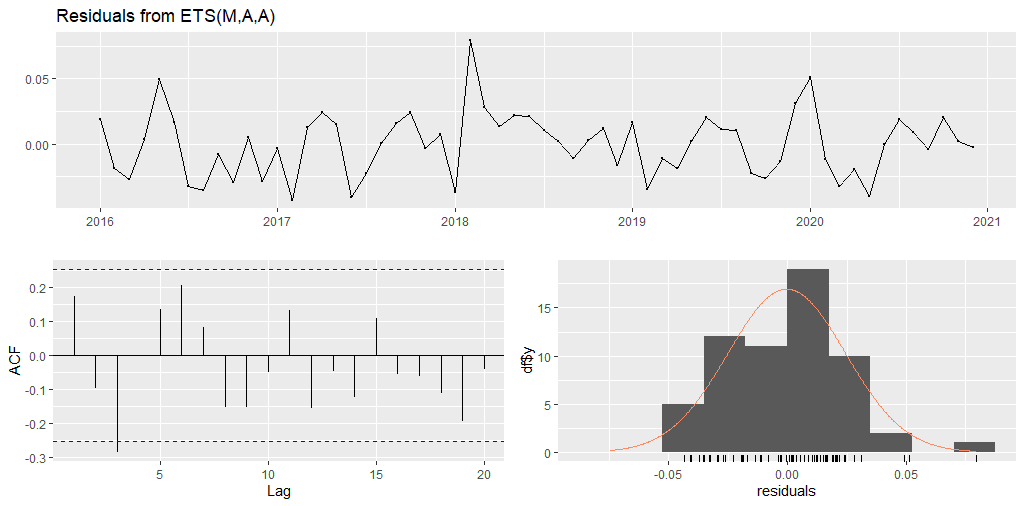


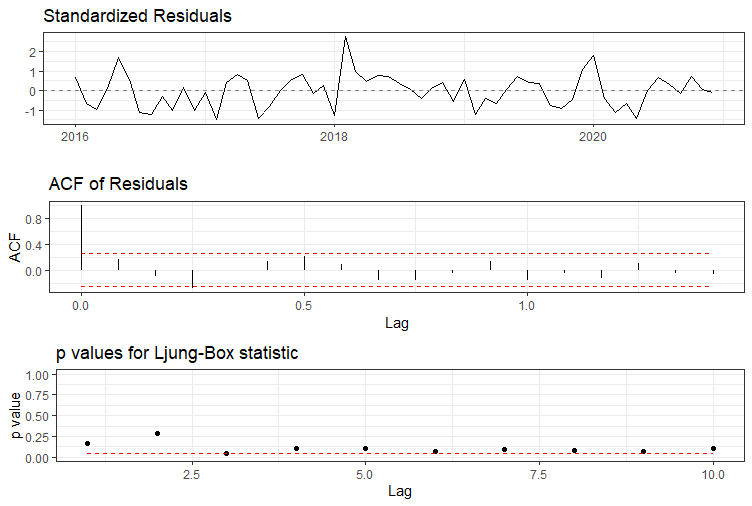


1. SARIMA-ETS hybrid model


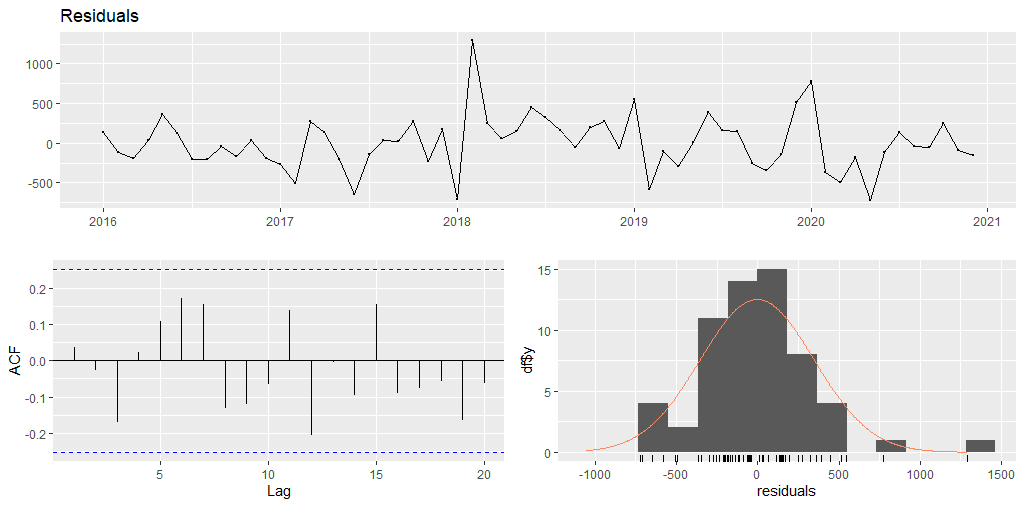


* Notably, a Ljung-Box test is not available from the “ggfortify” package.
