# Supplementary material for: Forecasting of Milk Production in Northern Thailand Using Seasonal Autoregressive Integrated Moving Average, Error Trend Seasonality, and Hybrid Models
Source: Front Vet Sci. 2021 Nov 30;8:775114. doi: 10.3389/fvets.2021.775114 (PMC8669476; doi:10.3389/fvets.2021.775114)
Supplement: Supplementary file 2 [file Data_Sheet_2.docx]

**Supplementary S2**

Milk production forecast from Seasonal Autoregressive Integrated Moving Average (SARIMA), Error Trend and Seasonality (ETS) and SARIMA-ETS models for the northern region of Thailand.

| Date | SARIMA | ETS | Hybrid |
| --- | --- | --- | --- |
| 1/1/2021 | 16304.93 | 15706.92 | 16005.93 |
| 1/2/2021 | 15721.18 | 15752.74 | 15736.96 |
| 1/3/2021 | 17248.77 | 17382.88 | 17315.83 |
| 1/4/2021 | 16588.53 | 16748.82 | 16668.68 |
| 1/5/2021 | 16370.51 | 16627.51 | 16499.01 |
| 1/6/2021 | 15691.23 | 15583.06 | 15637.14 |
| 1/7/2021 | 15347.76 | 15114.6 | 15231.18 |
| 1/8/2021 | 14453.26 | 14239.78 | 14346.52 |
| 1/9/2021 | 13420.55 | 13463.02 | 13441.78 |
| 1/10/2021 | 14240.67 | 14193.98 | 14217.33 |
| 1/11/2021 | 14629.84 | 14641.39 | 14635.61 |
| 1/12/2021 | 16141.39 | 15897.22 | 16019.31 |
| 1/1/2022 | 16962.55 | 16170.38 | 16566.46 |
| 1/2/2022 | 16333.38 | 16216.2 | 16274.79 |
| 1/3/2022 | 17697.28 | 17846.34 | 17771.81 |
| 1/4/2022 | 17085.76 | 17212.28 | 17149.02 |
| 1/5/2022 | 16704.66 | 17090.96 | 16897.81 |
| 1/6/2022 | 16063.68 | 16046.51 | 16055.1 |
| 1/7/2022 | 15795.33 | 15578.06 | 15686.7 |
| 1/8/2022 | 14877.2 | 14703.24 | 14790.22 |
| 1/9/2022 | 13903.24 | 13926.48 | 13914.86 |
| 1/10/2022 | 14832.89 | 14657.43 | 14745.16 |
| 1/11/2022 | 15169.02 | 15104.84 | 15136.93 |
| 1/12/2022 | 16528.42 | 16360.68 | 16444.55 |
